# A facile method to generate cerebral organoids from human pluripotent stem cells

**DOI:** 10.17179/excli2023-6299

**Published:** 2023-10-05

**Authors:** Susan Simorgh, Seyed Ahmad Mousavi, San Kit To, Vincent Pasque, Keimpe Wierda, Tim Vervliet, Meghdad Yeganeh, Paria Pooyan, Yoke Chin Chai, Catherine Verfaillie, Hossein Baharvand

**Affiliations:** 1 Department of Developmental Biology, School of Basic Sciences and Advanced Technologies in Biology, University of Science and Culture, Tehran, Iran; 2 Department of Stem Cells and Developmental Biology, Cell Science Research Center, Royan Institute for Stem Cell Biology and Technology, ACECR, Tehran, Iran; 3 Stem Cell Institute, Department of Development and Regeneration, KU Leuven, Leuven 3000, Belgium; 4 Department of Development and Regeneration, Lab for Epigenetic Reprogramming, Leuven Stem Cell Institute, Leuven Single-Cell Omics Institute and Leuven Cancer Institute, KU Leuven-University of Leuven, Leuven 3000, Belgium; 5 VIB-KU Leuven Center for Brain & Disease Research, Leuven 3000, Belgium; 6 Electrophysiology Unit, Leuven 3000, Belgium; 7 Laboratory of Molecular and Cellular Signaling, Department of Cellular and Molecular Medicine, KU Leuven, Leuven 3000, Belgium

**Keywords:** cerebral organoids, human pluripotent stem cell, hanging drop, neural induction protocol, single nuclei RNA-sequencing analysis

## Abstract

Human cerebral organoids (COs) are self-organizing three-dimensional (3D) neural structures that provide a human-specific platform to study the cellular and molecular processes that underlie different neurological events. The first step of CO generation from human pluripotent stem cells (hPSCs) is neural induction, which is an *in vitro* simulation of neural ectoderm development. Several signaling pathways cooperate during neural ectoderm development and *in vitro* differentiation of hPSCs toward neural cell lineages is also affected by them. In this study, we considered some of the known sources of these variable signaling cues arising from cell culture media components and sought to modulate their effects by applying a comprehensive combination of small molecules and growth factors for CO generation. Histological analysis demonstrated that these COs recapitulate the neural progenitor zone and early cortical layer organization, containing different types of neuronal and glial cells which was in accordance with single-nucleus transcriptome profiling results. Moreover, patch clamp and intracellular Ca^2+^ dynamic studies demonstrated that the COs behave as a functional neural network. Thus, this method serves as a facile protocol for generating hPSC-derived COs that faithfully mimic the features of their *in vivo* counterparts in the developing human brain.

See also Figure 1(Fig. 1).

## Introduction

The human brain is an intricate organ, at both the cell architecture and functional level. A highly ordered layered organization of about 86 billion neurons alongside about 85 billion glial cells are working together in a dynamic network, comprised of innumerable synapses (Herculano-Houzel, 2009[15]; Von Bartheld et al., 2016[55]). Surprisingly, this extent of complexity in brain structure and function originates from a primary hollow neural tube, lined with neural progenitor cells, which give rise to all different types of neuronal and glial cells during development (Kelley and Pașca, 2022[21]). The current insights into central nervous system development have been fundamentally obtained through rodent model studies. However, there are multiple human-specific features in brain cellular composition and function that make it difficult and inaccurate to interpret the rodent model study results in the human context (Chiaradia and Lancaster, 2020[7]). Human brain research has mainly relied on tissues from aborted fetuses, postmortem samples, and surgical biopsies from patients. However, there is limited access to these tissues and they present only a narrow temporal shot of developmental or pathological events, and there are no human models to investigate the dynamics of cellular and molecular processes involved *in* neurodevelopment or neurological diseases (Eichmüller and Knoblich, 2022[11]).

The advent of human pluripotent stem cells (hPSCs) (Takahashi et al., 2007[48]; Thomson et al., 1998[51]) with their inherent potential for *in vitro* generation of most cell types of the body, has provided a unique platform to study human-specific biological and medical features. Over the last two decades, a multitude of protocols has been published on the generation of different neural cells in both two-dimensional (2D) and three-dimensional (3D) culture conditions (Kelava and Lancaster, 2016[20]). Although 2D culture enables studying some of the features of developing brain cells, the emergence of 3D cell culture methods, such as organoids, also enables studying the interaction between different types of neural cells as well as the intricate architecture development in the brain tissue (Kretzschmar and Clevers, 2016[24]). The hPSC-derived cerebral organoids (COs) fulfill the criteria defined for organoids, including 3D self-organization into a brain-like cytoarchitecture, comprised of diverse neural and in some cases non-neural cell types, displaying brain-like electrophysiological activity (Pașca et al., 2022[32]). In addition, as they can be maintained in 3D cell culture for many months, they progressively reach the late fetal or early postnatal maturation levels (Gordon et al., 2021[13]; Lancaster and Knoblich, 2014[26]). Since 2013, numerous articles have been published on neural organoid generation methods from hPSCs. All these studies demonstrated that neural organoids present a faithful, albeit reductionist model, mirroring the cellular organization, composition, and function of different parts of the nervous system (Paşca et al., 2015[33]; Qian et al., 2016[37]; Renner et al., 2017[40]; Velasco et al., 2019[53]; Watanabe et al., 2017[57]).

However, the differentiation outcome of hPSCs-derived COs obtained from different established protocols has exhibited degrees of variability in the obtained neural cell fates and composition (Kim et al., 2021[22]; Quadrato et al., 2017[39]; Velasco et al., 2019[53]). The sources of these variations in neural organoid generation outcomes have been addressed in some studies. Batch-to-batch heterogeneity of cell culture media components such as serum, knockout serum replacement (KOSR), B-27 supplements, and commercial extracellular matrices like Matrigel, can affect the efficiency of neural induction from hPSCs (Aisenbrey and Murphy, 2020[1]; Kozlowski et al., 2021[23]; Nasu et al., 2012[30]; Tchieu et al., 2017[50]). Furthermore, hPSC line-dependent variations in the endogenous signaling pathways, mainly WNT signaling, have been demonstrated to affect the result of neural induction (Strano et al., 2020[46]). In addition, some technical considerations such as hPSC passage number and the initial cell density also can act on the efficiency of differentiation (Lancaster et al., 2017[25]; Qian et al., 2018[36]). To overcome this variation challenge, several approaches have been pursued in different studies, e.g., using chemically defined media, like Essential 8 (E8) for hPSC culture and Essential 6 (E6) for neural induction (Yoon et al., 2019[60]), testing different batches or lot numbers of cell culture materials, like KOSR, to find the batches which are suitable for neural cell fate induction, and also applying different combinations of small molecules and growth factors to guide the hPSCs differentiation toward the desired neural cell fate while blocking other fates.

In this study, we sought to apply some modifications to develop a more facile protocol for CO generation from hPSCs. We first explored the published CO generation methods and their results in detail. Based on the studies by two pioneer groups (Kadoshima et al., 2013[18]; Lancaster et al., 2013[27]), these methods can be categorized into "guided" and "unguided" protocols. The unguided method is based on the "Default" model of neural induction, which implies that the default choice of embryonic epiblast is the neuroectoderm fate and is determined by intrinsic factors e.g., Zfp521 transcription factor, while the epiderm and endomesodermal fates should be induced via extrinsic signaling pathways (Hemmati-Brivanlou and Melton, 1997[14]; Kamiya et al., 2011[19]; Spemann and Mangold, 2003[44]; Wilson and Hemmati-Brivanlou, 1995[58]). As the in vitro differentiation of PSCs recapitulates the embryonic events, the default fate of PSC differentiation is also neural ectoderm. In other words, PSCs differentiate to the neural fate if they do not receive any extrinsic signaling cues to guide them toward other possible cell fates. But practically speaking, signaling molecules are always present in the *in vitro* differentiation of PSCs, arising from cell culture media components or endogenously originated from differentiating PSCs. Therefore, the outcome of the unguided method for CO generation can be inconsistent (Quadrato et al., 2017[39]; Velasco et al., 2019[53]). In contrast, the guided protocols employ small-molecule treatments to guide hPSCs toward the neural fate and block alternative cell fate pathways during the neural induction step (Kadoshima et al., 2013[18]; Paşca et al., 2015[33]; Qian et al., 2016[37]).

Different published protocols for CO generation display some strong points as well as some drawbacks in their method. Here, we devised a straightforward method with a modified combination of small molecules and growth factors that covers different advantageous features to aid the differentiation of COs from hPSCs. These COs comprised different types of neural progenitors, functional neurons, astrocytes, and oligodendrocyte progenitor cells and can be potentially employed as *in vitro* model to study cell composition, architecture, and function of the human embryonic brain. 

## Materials and Methods

### Culture of human pluripotent stem cells

Three human pluripotent stem cell (hPSC) lines were used in this study. The hESC line H9 (WA09, purchased from the WiCell Research Institute, female, RRID: CVCL_9773), the hiPSC line ChiPSC6b (purchased from Cellectis/Cellartis, male, RRID: CVCL_UK34), and the hiPSC line SIGi001-A (iPSC EPITHELIAL-1, IPSC0028, purchased from Sigma, female, RRID: CVCL_EE38). hPSC lines were cultured on hESC-qualified Matrigel (BD Biosciences) in Essential 8 flex medium (Thermo Fisher Scientific). Every 4-5 days and after reaching 75 % confluency, the hPSCs were passaged with 0.5 mM EDTA solution dissolved in PBS (Gibco, Thermo Fisher Scientific) in a 1:3 split ratio. All cell cultures were regularly tested and confirmed negative for mycoplasma contamination and maintained in 5 % CO_2_ incubators at 37 °C. The passage number of all hPSC lines was below 50 during the experiments.

### Generation of cerebral organoids

For COs generation, hPSCs were trypsinized to make a single cell suspension in the neural induction medium (NIM), containing DMEM/F-12, 20 % (vol/vol) KnockOut Serum Replacement (KOSR), 1 % (vol/vol) Insulin-Transferrin-Selenium (ITS), 1 % (vol/vol) GlutaMAX, 1 % (vol/vol) MEM-NEAA, 1 % (vol/vol) sodium pyruvate, 1 % (vol/vol) penicillin-streptomycin, 0.1 % (vol/vol) 2-Mercaptoenthanol (all from Gibco, Thermo Fisher Scientific). For aggregate formation on day 0, the hanging drop method was used, and the NIM was supplemented with 1 % (vol/vol) methylcellulose (Sigma), 50 μM ROCK inhibitor Y27632 (Sigma), 10 μM SB431542 (Tocris), and 3 μM IWR-1-endo (Merck Millipore/Sigma). Single droplets comprising 25-30 μL of day 0 medium and 20,000 singularized hPSCs, were put onto the inside part of a turned-over lid of a cell culture plate. After 24 hours, on day 1, the drops, containing formed aggregates, were collected using a P1000 tip and transferred into a 60 mm non-treated cell culture dish (Corning). The aggregates were washed once with DMEM/F-12 to remove the day 0 medium then the NIM was added, supplemented with 10 μM SB431542 (Tocris), 3 μM IWR-1-endo (Merck Millipore/Sigma), 2.5 μM dorsomorphin (Sigma), and 200 nM LDN-193189 (Miltenyi Biotec). From day 1 onward until the end of the experiment, the cell culture dishes were placed on an orbital shaker (Fisher Scientific, 15689415) rotating at 80 RPM. On day 4 the medium was refreshed with NIM supplemented with 2.5 μM dorsomorphin (Sigma), and 2 μM A83-01 (Tocris). 

From day 7, the medium was changed to neural proliferation medium (NPM), containing a 1:1 mixture of DMEM/F12 and Neurobasal A medium, 2 % (vol/vol) B27 supplement without vitamin A, 1 % (vol/vol) Insulin-Transferrin-Selenium (ITS), 0.5 % (vol/vol) N2 supplement, 1 % (vol/vol) GlutaMAX, 0.5 % (vol/vol) MEM-NEAA, 1 % (vol/vol) penicillin-streptomycin, 1 % (vol/vol) chemically defined lipid concentrate (CDLC), 0.1 % (vol/vol) 2-Mercaptoenthanol (all from Gibco, Thermo Fisher Scientific), 1 μg ml^-1^ heparin (Sigma), 20 ng ml^-1 ^ Fibroblast growth factor 2 (FGF2, Peprotech), and 20 ng ml^-1 ^epidermal growth factor (EGF, R&D Systems). On day 7, organoids were fed with NPM supplemented with 3 μM CHIR99021 (Tocris), 2 μM A 83-01 (Tocris), and 10 μg ml^-1^ heparin (Sigma). From day 10 to 25, the organoids continued to grow in NPM and the medium was replaced every other day.

To promote neural differentiation, after day 25, the medium was altered to neural maturation medium (NMM), containing a 1:1 mixture of DMEM/F12 and Neurobasal A medium, 2 % (vol/vol) B27 supplement, 0.5 % (vol/vol) N2 supplement, 1 % (vol/vol) GlutaMAX, 0.5 % (vol/vol) MEM-NEAA, 1 % (vol/vol) penicillin-streptomycin, and 0.1 % (vol/vol) 2-Mercaptoenthanol (all from Gibco, Thermo Fisher Scientific). From day 25 to 60, the organoids were fed every other day, by NMM supplemented with 20 ng ml^-1 ^brain-derived neurotrophic factor (BDNF, Peprotech), 20 ng ml^-1 ^glial-derived neurotrophic factor (GDNF, Peprotech), and 1 μg ml^-1^ heparin (Sigma). From day 60 until the end of the experiment, the NMM was replaced in half volume every 2-3 days and supplemented with 10 ng ml^-1 ^brain-derived neurotrophic factor (BDNF, Peprotech), 10 ng ml^-1 ^glial-derived neurotrophic factor (GDNF, Peprotech), 1 μM dibutyryl-cAMP (cAMP, Stemcell Technologies), and 200 μM L-Ascorbic acid (AA, Sigma).

### Histological preparation

On the sampling days, the COs were picked up randomly using a cut-end P1000 tip and washed twice with PBS, then fixed with 4 % paraformaldehyde (PFA) overnight at 4 °C. After two times washing with PBS, COs were transferred to 30 % sucrose (Acros Organics) and kept at 4 °C until fully sunk into the bottom of the sucrose solution tube. Afterward, COs were molded into an embedding medium (Tissue-Tek optimum cutting temperature (OCT) compound, Sakura), then snap-frozen on dry ice and kept at -80 °C or -20 °C. Organoids were cut into 20 μm sections with a cryostat (CryoStar NX70). Cryosections were mounted onto Superfrost Plus slides (Fisher Scientific) and stored at -80 °C or -20 °C.

### Immunofluorescence staining

Cryosectioned slides were dried at room temperature (RT) for 15 minutes (min). To remove the OCT and rehydration, the slides were washed with PBS, three times, 10 min for each wash. To perform Heat-mediated antigen retrieval, the slides were immersed in a heat-resistant container filled with 1x Target Retrieval Solution (Dako) and steamed for 20 min, in a food steamer. After washing with 0.05 % PBST (PBS-Tween20, Sigma), the slides were permeabilized and blocked in 0.5 % PBS-TritonX100 (Sigma) containing 5 % donkey or goat serum (Dako), for 1 hour at RT. Slides were incubated with primary antibodies (Supplementary Table 1), diluted with 5 % serum in PBST, overnight at RT. Following three times of PBST washing (10 min/wash), the secondary antibodies (Supplementary Table 1), diluted with 5 % serum in PBST, were applied and incubated for 1 hour at RT, then washed three times with PBST. Nuclei were counterstained with Hoechst 33342 solution (Sigma, dilution 1:1,000) and coverslips were mounted on the stained slides using mounting media (ProLong Gold Antifade Reagent, Thermo Fisher Scientific).

A confocal microscope (Nikon C2) was used for image acquisition and sample images were processed with ImageJ software (Fiji).

### Nuclei isolation from cerebral organoids and single nucleus RNA-sequencing (snRNA-seq) analysis

For each sample, about 4-5 COs were pooled together and the nuclei isolation was performed using a protocol previously described in Thrupp et al. (2020[52]). In brief, the samples were homogenized using 15 gentle strokes via a douncer in 1 mL ice-cold HB buffer (Homogenization Buffer containing sucrose (320 mM), CaCl_2_ (5 mM), Mg(CH_3_COO)_2_ (3 mM), Tris (10 mM), EDTA (0.1 mM), NP-40 (0.1 %), 2-ME (1 mM), UltraPure water with RNase Inhibitor. The homogenates were filtered through a 70 μm strainer and centrifuged for 5 mins at 500 g and 4 °C. The pellet was resuspended in 2.65 mL of wash buffer and 2.65 mL of gradient medium, a final volume of 5.3 mL containing sucrose (320 mM), CaCl_2_ (5 mM), Mg(CH_3_COO)_2_ (3 mM), Tris (10 mM), EDTA (0.1 mM), 2-ME (1 mM), Optiprep (50 %), UltraPure water with RNase Inhibitor and complete protease inhibitor. The samples were layered over a 4 mL cushion that comprised 33 % Optiprep diluted in KCl (150 mM), MgCl₂ (30 mM), Tris (60 mM), Sucrose (250 mM), and water. The gradient columns were centrifuged using an SW41Ti rotor at 7700 rpm for 30 mins, at 4 °C. After removing the supernatant, the nuclei pellet was resuspended in PBS (1x), BSA (1 %), and RNase Inhibitor and strained through a 40 μm flowmi strainer. The nuclei suspension was counted with Luna-FL automated Fluorescence Cell Counter (Logos Biosystems).

To perform library preparation and sequencing, nuclei were loaded onto the 10X Chromium Single Cell Platform (10X Genomics) (Next GEM Single Cell 3' library and Gel Bead Kit v3.1) according to the manufacturer's protocol (10x User Guide; CG000204, Revision D). Generation of gel beads in emulsion (GEMs), barcoding, GEM-RT cleanup, complementary DNA amplification, and library construction were all performed according to the manufacturer's protocol. Individual sample quality was assessed using Tapestation (Agilent). Qubit 2.0 (ThermoFisher Scientific) was used for library quantification before pooling libraries. The final library pool was sequenced on the NextSeq2000 (Illumina) instrument using NextSeq 1000/2000 P3 kit v3 for 2 lanes of 100-base-pair paired-end reads.

The Cell Ranger version 6.1.1 was used to process, align, and summarize unique molecular identifier (UMI) counts against the 10X Genomics pre-built human GRCh38 reference genome datasets (2020-A, July 7, 2020). 

After gathering the snRNA-seq libraries of the two time points (day 80 and day 160), the Cell Ranger outputs were analyzed by Seurat (v4.0) in the R (v4.1.1) framework. Before preparing the cells for global clustering, all cells containing more than 1500 detected genes and genes that were expressed in at least three cells were kept. Since day 80 consisted of three pooled samples from three different hPSC lines (ChiPSC6b, H9, and SIGi001-A) and day 160 consisted of two samples (ChiPSC6b and H9), the data had to be demultiplexed first. We used scSplit to demultiplex each day based on SNP information. The scSplit with default parameters and hg38 as reference genome was applied to get the list of expressed cell IDs in each corresponding cell line. Then, the scSplit output information was applied on Seurat objects for each timepoint.

After demultiplexing, we integrated two time points of each cell line, using the Seurat Integration procedure with default parameters. Then clustering was performed for integrated cell lines objects (Day 80 + Day 160) and SIGi001-A cell line object (Day 80). Differentially expressed genes were determined by top markers found in each cluster with adjusted P-value < 0.05 and positive fold change of 1.20 and above. The violin plots and feature plots were designed from clustering Seurat objects.

### Electrophysiological assessments

Human COs were removed from culture medium and quickly embedded in 2 % low-melting agarose (Sigma-Aldrich, A0576) diluted in PBS (in mM: 137 NaCl, 2.7 KCl, 10 Na_2_PO_4_, 1.8 KH_2_PO_4_, pH= 7.4). Dissolved agarose was kept in a water bath at 40 °C until use. Embedding was done on ice to quickly solidify the agarose gel and subsequently sectioned using a Leica VT1200S vibratome (amplitude: 0.3-0.5 mm, velocity: 0.07-0.9 mm/sec) in ice-cold oxygenated (95 % O_2_ and 5 % CO_2_) artificial cerebrospinal fluid (aCSF), containing (in mM): 127 NaCl, 25 NaHCO_3_, 1.25 NaH_2_PO_4_, 2.5 KCl, 1 MgCl_2_, 2 CaCl_2_, and 25 glucose and subsequently stored in a holding chamber at room temperature. For recordings the slices were continuously perfused in a submerged chamber (Warner Instruments) at a rate of 3- 4 ml/min with oxygenated aCSF at a temperature of 33 ± 1 °C. Internal whole cell patch solution contained (in mM): 135 K-gluconate, 4 KCl, 10 HEPES, 4 Mg-ATP, 0.3 Na_2_-GTP, 10 phosphocreatine and biocytin (3 mg/ml, pH = 7.3, ~295 mOsm). Whole-cell patch-clamp recordings were done using borosilicate glass recording pipettes (resistance 3.5-5 MΩ, Sutter P-1000), using a double EPC-10 amplifier under control of Patchmaster v2 x 32 software (HEKA Elektronik, Lambrecht/Pfalz, Germany). Currents were recorded at 20 Hz and low pass filtered at 3 kHz when stored. The series resistance was compensated to 70-75 %. Cell intrinsic properties were recorded in current clamp, while sEPSCs and I/V curves were recorded in voltage clamp (V_hold_= -70 mV). I/V-traces were generated using a step protocol (-80 to +20 mV, 10 mV steps, 200 ms). After resting membrane potential (Vm) was measured in current clamp (I holding = 0 pA), the cells were kept at ~ -70 mV during experiments involving evoked action potential initiation. Immediately after recordings, the slices were fixed in 4 % PFA. Cell intrinsic properties were analyzed using Fitmaster (HEKA Elektronik, Lambrecht/Pfalz, Germany) and sEPSC input was analyzed using Mini Analysis program (Synaptosoft).

### Fluo-4 AM-mediated intracellular Ca^2+^ imaging

Loading and de-esterification steps were performed in a humidified incubator at 37 °C and 5 % CO_2_. COs were incubated/loaded with 1 µM Fluo-4 AM solubilized in neural maturation medium (NMM). Next, the organoids were washed twice with NMM and then transferred to poly-L-lysine coated plates and de-esterification was allowed to occur for 45 min in NMM. Just before starting the Ca^2+^ imaging experiments, the organoids were spun down at 500 g for 5 min to improve attachment to the culture plates. Then the NMM was replaced with a pre-warmed (37 °C) modified Krebs-Ringer solution (135 mM NaCl, 6.2 mM KCl, 1.2 mM MgCl_2_, 12 mM HEPES (pH 7.3), 11.5 mM glucose and 2 mM CaCl_2_). KCl addition was performed through a continuous perfusion to prevent detachment of the organoid. For the KCl stimulus the modified Krebs-Ringer solution was prepared substituting the NaCl for 140 mM KCl. Imaging was performed using a Nikon eclipse Ti2 inverted fluorescence microscope (Nikon) equipped with excitation filter FF01-378/474/554/635, dichroic mirror FF01-432/515/595/730 and emission filter 515/30 all from Semrock. Excitation was performed at 470 nm using a Coolled pR-4000 (Coolled). Acquisition of the fluorescent signal at 520 nM was performed at 3 Hz using a pco.edge 4.2bi sCMOS camera (pCO). For analysis the FIJI software was utilized. Background was subtracted from all images, after which regions of interest were determined where spontaneous or agonist induced changes in Ca^2+^ indicator intensity was observed. 

### Statistical analysis

Data are presented as the mean ± SD. Statistical analyses were performed using two-way or one-way ANOVA test, as indicated in figure legends, in GraphPad Prism version 8.0.0 for Windows, GraphPad Software, San Diego, California USA, www.graphpad.com. Significant differences were defined by P values, P < 0.05 (*), P < 0.01 (**), P < 0.001 (***), and P < 0.0001 (****), as indicated in the figure legends.

## Results

### Optimizing the cerebral organoid generation method 

**Figure 2:**
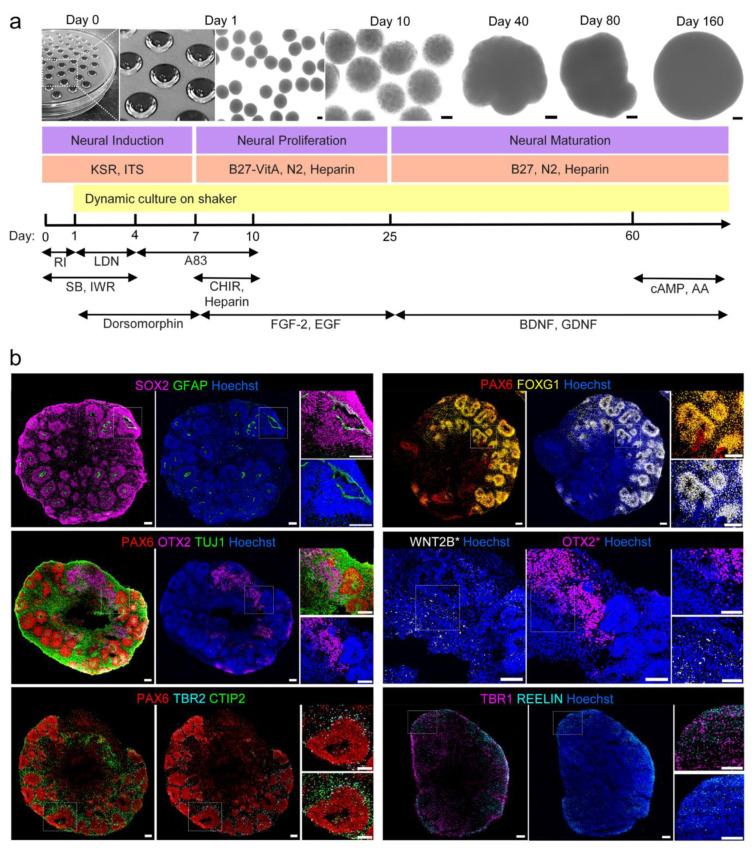
Human pluripotent stem cell-derived cerebral organoids simulate cell types and organization of the developing human brain. (a) Diagram of differentiation protocol and bright-field images of COs on different time points. (RI: ROCK Inhibitor Y27632, LDN: LDN193189, SB: SB431542, IWR: IWR-1-endo, A83: A83-01, CHIR: CHIR99021, FGF-2: Fibroblast growth factor 2, EGF: Epidermal growth factor, BDNF: Brain derived neurotrophic factor, GDNF: Glial cell derived neurotrophic factor, cAMP: Cyclic adenosine monophosphate, AA: Ascorbic acid); (b) Immunostaining of COs at day 40 to illustrate ventricular zone (VZ)-like structures (SOX2/GFAP), Dorsal forebrain identity (PAX6/FOXG1), cortical hem-like region (PAX6/TUJ1/OTX2 and WNT2B*), early cortical layers formation (PAX6 for VZ, TBR2 for SVZ, CTIP2 and TBR1 for cortical neuronal layers V and VI, REELIN for the marginal zone). * WNT2B and OTX2 staining were not performed on the same cryosection. We selected a close adjacent slice to perform WNT2B staining and compare it to the OTX2 expression pattern. All the scale bars are 100 μm.

In most studies, 96-well ultra-low attachment plates with a round (U or V) shaped bottom (Kadoshima et al., 2013[18]; Lancaster et al., 2013[27]) or Aggrewell plates (Yoon et al., 2019[60]) are used to promote cell aggregation of singularized hPSCs. Here, we used a modified hanging drop technique as a readily feasible and cost-effective method, to efficiently generate hPSCs aggregates. In the conventional hanging drop method, the cell aggregation process takes up to 2 or 3 days. In this study, we added 1 % methylcellulose to increase viscosity of the hanging drop medium and achieve accelerated hPSC aggregate formation. Consequently, hPSCs aggregate formation occurred after only 24 hours, resulting in uniformed smooth-edge spherical structures on day 1 (Figure 2a(Fig. 2)). Similar methods have been used for cancer cell line aggregate formation by adding a higher concentration (20 %) of methylcellulose to the drops or round-bottom wells (Ware et al., 2016[56]; Cavo et al., 2020[4]).

Starting from day 1, aggregates were transferred to polystyrene non-treated culture dishes and cultures were continued under dynamic condition on an orbital shaker (Figure 2a(Fig. 2)). During the first 7 days, we added a mixture of small molecules composed of IWR-1, as WNT signaling pathway inhibitor, SB-431542 and A83-01 to inhibit TGFß signaling pathways, in addition to LDN-193189 and dorsomorphin to inhibit BMP signaling pathways. This combination of small molecules should drastically counteract the possible effect of interfering signaling pathways to achieve more guided neural induction (Chambers et al., 2009[5]; Surmacz et al., 2012[47]; Vogt et al., 2011[54]; Zhou et al., 2010[64]). 

Most CO protocols have used Matrigel to promote formation of ventricular zone-like (VZ-like) structures. However, to remove the variable batch effects of Matrigel, we omitted embedding of organoids into Matrigel droplets and also did not add soluble Matrigel to the culture medium. Because boosting WNT signaling promotes the expansion of VZ-like structures (Lancaster et al., 2017[25]), the COs were exposed to the WNT activator, CHIR99021, between day 7 and 10, along with neural proliferation medium containing bFGF, EGF and heparin, until day 25. The VZ-like structures were properly formed within COs around day 30 and contained neural progenitor cells displaying a radial organization surrounding a ventricular cavity-like space (Figure S1a; see supplementary information). Considering the role of heparin as an anchoring site for different growth factors, a WNT signaling amplifier, and interacting with different extracellular matrix (ECM) glycoproteins (Bejoy et al., 2018[3]; Colombres et al., 2008[8]; Peall et al., 2021[34]; Yu et al., 2017[61]), we continued heparin treatment until day 60, in combination with neural maturation medium containing BDNF and GDNF.

We used three hPSC lines to generate COs, including one human embryonic stem cell (hESC) line WA09/H9*,* and two human induced pluripotent stem cell (hiPSC) lines, ChiPSC6b and SIGi001-A (Figure S1b). All the cell lines demonstrated homogenous aggregate formation on day 1, with an average size of 350-450 µm (Figure S1c). The early appearance of radial arrangements of neural progenitor cells was evident from day 10, both inside and at the periphery of the organoids, and complete formation of VZ-like structures was observed between days 35 to 40. The COs were maintained in culture for 160 days. However, as has also been reported for some other cell lines in other studies (Yoon et al., 2019[60]), COs from the SIGi001-A iPSC line began to disintegrate around day 90. 

### Exploring the cell fates and organization within cerebral organoids

Immunohistology assessment of SOX2 and GFAP, as specific markers for radial glial neural progenitor cells, in COs on day 40 (Figure 2b(Fig. 2)) revealed several VZ-like architectures in the organoids. These cellular structures also expressed FOXG1 and PAX6, demonstrating that the COs acquired an anterior-dorsal neural identity. Presence of WNT2B expression adjacent to OTX2-positive cells, demonstrated presence of the cortical hem. We also detected TBR2-positive intermediate progenitor cells localized outside the VZ-like (PAX6^+^) domains, as well as CTIP2, TBR1, and REELIN-expressing neural cells, respectively representing the subventricular zone-like (SVZ) and early cortical plate and marginal zone layer formation within COs. It is worth noting that the COs derived from different hPSC lines exhibited similar patterns of VZ-like organization, early cortical layer formation and also neural cell fate composition (Figure S2a, S2b).

### Tracking the temporal maturation events in cerebral organoids

**Figure 3:**
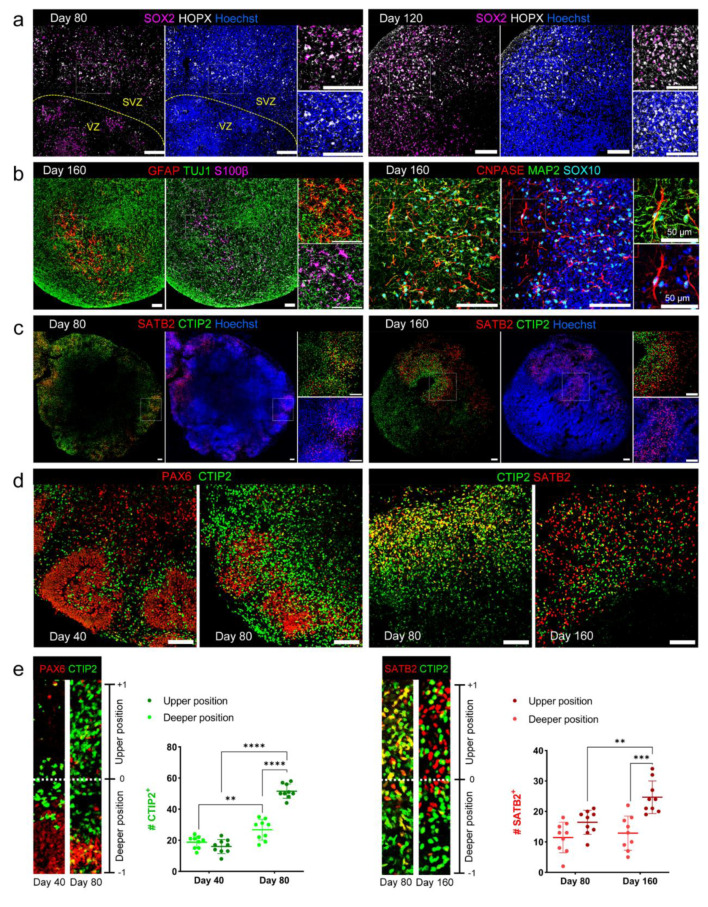
Progressive increase in cerebral organoids maturation. (a) Immunostaining for SOX2 and HOPX markers at days 80 and 120 COs demonstrated the presence of bRG cells in the SVZ-like region, just next to the VZ-like progenitor region, SOX2^+^/ HOPX^- ^(separated by the yellow dashed line). (VZ: ventricular zone, SVZ: subventricular zone, bRG: basal radial glial). (b) COs immunostained for astrocytes (GFAP/S100β) and oligodendrocytes (CNPASE/SOX10) markers at day 160, revealing the presence of glial cells beside to neuronal population (TUJ1, MAP2). (c) Immunostaining of COs at days 80 and 160, for CTIP2/SATB2 as deep and upper layer cortical neuron markers, showing the spatiotemporal pattern of their emergence. (d, e) Quantification of CTIP2^+^ deep layer cortical neurons expansion between day 40 to day 80. Quantification of SATB2^+^ upper layer cortical neurons expansion between day 80 to day 160. (All data represented as mean ± SD; n=9 bins from 4 COs; two-way ANOVA, **P < 0.01, ***P < 0.001, ****P < 0.0001). All the scale bars are 100 μm.

Co-expression of HOPX^+^ and SOX2^+^ indicated the presence of basal radial glial (bRG) cells, also called outer radial glial (oRG) cells, in the COs, beginning from about day 80 and becoming more prominent on day 120 (Figure 3a(Fig. 3)). These bRG cells were located outward from the VZ-like region (HOPX-/SOX2^+^), mimicking the outer SVZ location, which is a primate-specific feature of the developing brain and the underlying attributor for the more expanded cortical layers in primates compared to rodents. Another indicator of progressive neural maturation is the gliogenic switch in the program of neural cell production. In fact, during human brain development, astrocyte and oligodendrocyte generation commences between gestational weeks 13 to 16 (Yang et al., 2022[59]). Consistently, we detected GFAP and S100*β* expressing astrocytes as well as CNPASE and SOX10 expressing oligodendrocytes amongst the neuronal populations, marked with TUJ1 and MAP2 expression, in day 160 COs (Figure 3b(Fig. 3)).

Furthermore, the spatiotemporal pattern of SATB2 expression, as an upper-layer cortical neuron marker, was reflecting the inside-early, outside-late pattern of cortical neurogenesis (Molnár et al., 2019[28]). We demonstrated that SATB2^+^ upper-layer neurons became evident from about day 80, whereas CTIP2^+^ deep-layer cortical neurons were present as early as day 40 in COs, and the production of both subtypes progressively increased over time (Figure 3c, 3d(Fig. 3)). To quantify the spatial distribution of CTIP2 and SATB2 expressing cortical neurons, we considered each of the VZ-like structures as a cortical neuron production unit and defined relative positional values in putative bins. This analysis indicated the upper position is preferentially more occupied at later time points. In other words, on day 80 compared to day 40, there are more CTIP2^+^ neurons within the upper positions of cortical bins, and also more SATB2^+^ neurons in the upper position, on day 160 versus day 80 (Figure 3e(Fig. 3)). 

Different hPSC lines that were employed in this study revealed an almost similar display of maturation characteristics, such as bRG cells and upper layer cortical neurons exhibition on day 80 (Figure S3a), as well as astrocyte and oligodendrocyte emergence on day 160 (Figure S3b). However, it should be noted that fewer SATB2^+^ neurons were present in day 80 H9hESC-derived COs than in ChiPSC6b and SIGi001-A hiPSCs-derived COs. Nevertheless, on day 160 the H9hESC-derived COs showed increased expression of SATB2^+^ upper-layer neurons as in ChiPSC6b-derived COs. In addition, on day 160 COs, oligodendrocyte lineage cells were still at the progenitor stage. Although CNPASE^+^ oligodendrocytes were detected adjacent to MAP2^+^ neurons (Figure S3c), we could not detect any signal for the myelin basic protein (MBP) or other more mature oligodendrocyte-specific markers.

### Surveying transcriptomic signature of diverse single cells identity found within cerebral organoids

**Figure 4:**
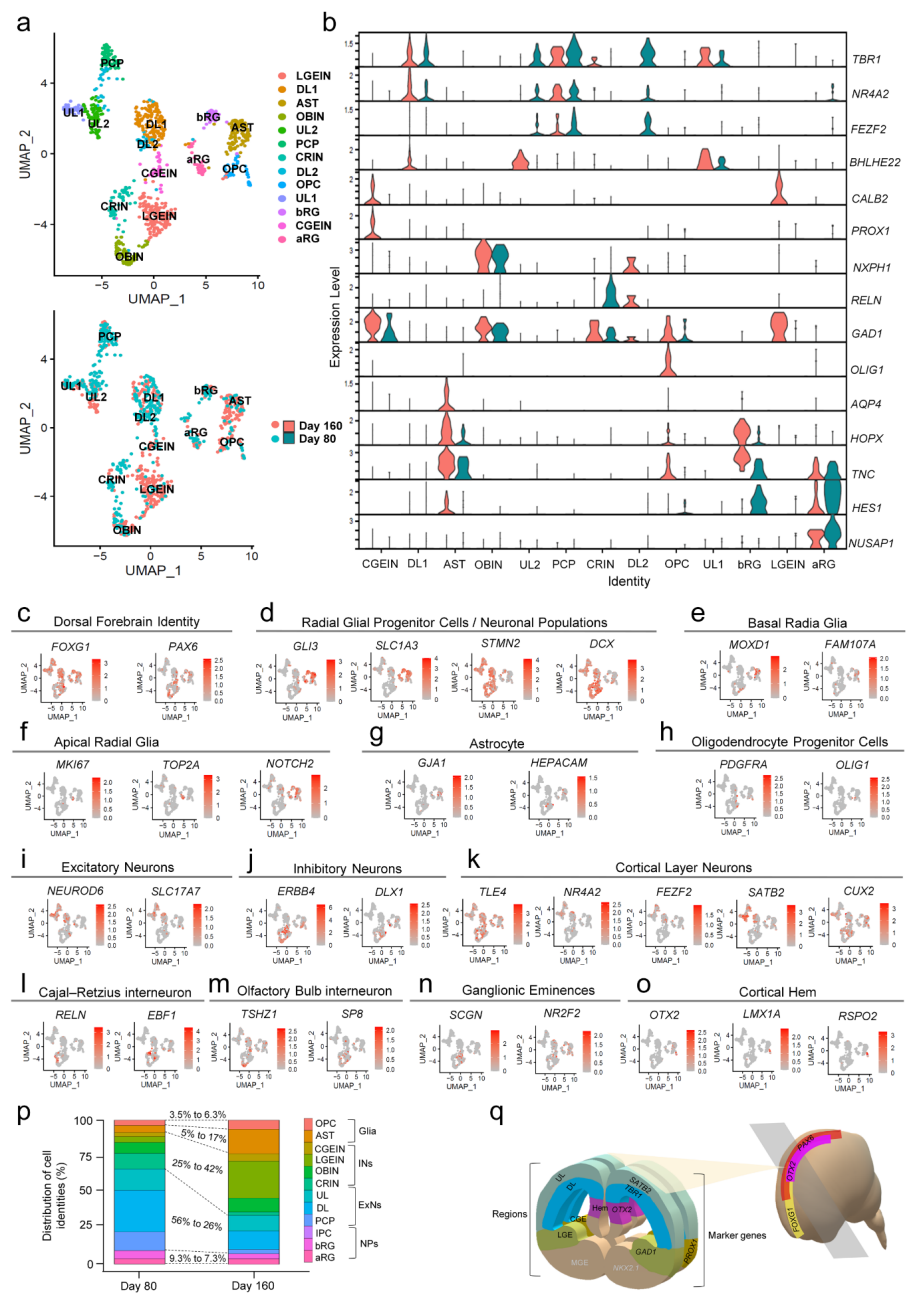
Characterization of different cell type composition in cerebral organoids using single nucleus RNA-sequencing analysis. (a) Uniform-manifold approximation map (UMAP) visualization of different cell-type clusters in COs derived from ChiPSC6b cell line. UMAPs are represented as integrated data from day 80 and day 160 with n=448 and n=476 nuclei analyzed for each day, respectively. Each dot represents a single nucleus from a single cell. In the upper UMAP, the color of each dot entitles the defined cell identities, and in the lower UMAP colors illustrate the corresponding day. (b) Violin plots demonstrating the expression level of selected specific markers to ascertain different cell identities in each cluster. (c-o) Individual UMAP plots, indicating the expression pattern of marker genes used to annotate a defined cell identity for each cluster. The gradient color shows the relative expression pattern from dark orange (higher expression) to light gray (lower expression). (p) Bar graphs illustrating the percentage of cellular distribution in different clusters, on days 80 and 160 COs. The colors label the cell type identities in the graphs. (q) Schematic representation of embryonic brain regions as the *in vivo* counterpart of the diverse neural cell fates found within COs. aRG: apical radial glial, bRG: basal radial glial, AST: astrocyte, OPC: oligodendrocyte progenitor cell, PCP: primary cortical plate, DL1,2: Deep layer cortical neuron 1,2, UL1,2: Upper layer cortical neuron 1,2, CGEIN: caudal ganglionic eminences interneuron, LGEIN: lateral ganglionic eminences interneuron, CRIN: Cajal-Retzius interneuron, OBIN: olfactory bulb interneuron, NPs: neural progenitor cells, IPC: intermediate progenitor cell, ExNs: excitatory neurons, INs: inhibitory neurons, MGE: medial ganglionic eminences)

To obtain a more in-depth characterization of different cell types within COs, we performed single-nucleus RNA sequencing (snRNA-seq) using the 10X Genomics Chromium platform. We analyzed a total of 2,602 single nuclei from COs derived from three hPSC lines, ChiPSC6b, H9, and SIGi001-A, and on two differentiation time points, days 80 and 160. To identify the differentially expressed genes in putative cell clusters, we used the unsupervised clustering method on integrated gene expression data sets from day 80 and 160 COs derived from ChiPSC6b (Figure 4(Fig. 4)) and H9 (Figure S4) cell lines, and only day 80 COs derived from SIGi001-A cell line (Figure S5). We used Uniform Manifold Approximation and Projection (UMAP) plots to visualize cell clusters (Figure 4a(Fig. 4) and S4a and S5a). To annotate cell type identities of each cluster, the differentially expressed genes were compared and matched to the known cell type-specific marker genes (Figure 4b(Fig. 4) and S4b and S5b). We found that almost all clusters expressed the indicator genes for anterior and dorsal neural identity, *FOXG1* and *PAX6*, respectively (Figure 4c(Fig. 4) and S4c and S5c). Also, no cells expressing ventral forebrain marker genes like *NKX2.1*, and no mesodermal (*TBXT*) and endodermal (*SOX17*) cell identities were detected. Therefore, the anterior-dorsal neural identity acquisition was confirmed on day 80 and 160 COs, in agreement with the immunofluorescence staining results on day 40. We also noticed a clear distinct localization between neuronal populations, expressing *STMN2* and *DCX*, and radial glial progenitor cell clusters, expressing *GLI3* and *SLC1A3* (Figure 4d(Fig. 4) and S4d and S5d). 

The radial glial progenitor cell population consisted of two distinct clusters. They expressed multiple genes e.g., *HES1*, *TNC*, *SLC1A3*, and *GLI3*, as a shared feature (Figure 4b,d(Fig. 4) and S4b,d and S5b,d). However, they were discernible in separate cell clusters: a basal radial glial (bRG) cell cluster, showing the molecular identity of *HOPX*, *FAM107A*, and *MOXD1* expression (Figure 4b,e(Fig. 4) and S4b,e and S5b,e) and an apical radial glial (aRG) cluster, containing cells expressing gene markers of proliferative cells, *NUSAP1*, *MKI67*, and *TOP2A*, along with *NOTCH2*, a gene expressed in the neural progenitor stage (Imayoshi et al., 2010[17]) (Figure 4f(Fig. 4) and S4f and S5f). The astrocyte (AST) and oligodendrocyte progenitor cell (OPC) clusters were evident on day 160, defined by the expression of *AQP4, GJA1, *and* HEPACAM* for astrocytes (Figure 4b,g(Fig. 4) and S4b,g and S5b,g), and *OLIG1* and *PDGFRA* for OPCs (Figure 4b,h(Fig. 4) and S4b,h and S5b,h).

The neuronal clusters were also categorized into two major classes of cortical neurons, glutamatergic excitatory projection neurons (*NEUROD6* and *SLC17A7) *(Figure 4i(Fig. 4) and S4i and S5i), and GABAergic inhibitory local circuit interneurons (*ERBB4 *and* DLX1*) (Figure 4j(Fig. 4) and S4j and S5j). Each of them included some distinct clusters related to different cell subtypes. The glutamatergic excitatory neurons appeared as three main clusters including primary cortical plate (PCP), deep layer (DL), and upper layer (UL) neurons. The primary cortical plate (PCP) cluster was mostly present on day 80 and contained neuronal cells that co-expressed gene markers for both deep and upper cortical layers, *TBR1* and *SATB2*, concomitantly with *FEZF2*, which is involved in determining the final fate and destination of primary cortical neurons (Chen et al., 2008[6]; Ozair et al., 2018[31]). We also differentiated the deep and upper layer cortical neuron clusters based on the expression level of *TBR1*, *TLE4*, and* NR4A2* in contrast to *SATB2*, *CUX2*, and *BHLHE22, *as indicator genes specifying the deep and upper cortical neurons, respectively (Figure 4k(Fig. 4) and S4k and S5k) (Doyle et al., 2021[10]; Fan et al., 2020[12]). The inhibitory interneurons were subdivided into 4 clusters, all of them expressing general interneuron gene markers, e.g., *GAD1* (Figure 4b(Fig. 4) and S4b and S5b). They could however be distinguished from each other based on the expression of *RELN *for Cajal-Retzius interneurons (CRINs) (Figure 4l(Fig. 4) and S4l and S5l), *TSHZ1* for olfactory bulb interneurons (OBINs) (Figure 4m(Fig. 4) and S4m and S5m), *SCGN, DLX, CALB2 *expression for both lateral and caudal ganglionic eminences (LGE, CGE), and higher expression of* PROX1 *and* NR2F2 (COUP-TFII)* for caudal ganglionic eminence (Figure 4b,n(Fig. 4) and S4b,n and S5b,n) (Schmitz et al., 2022[41]; Yu et al., 2021[63]). This indicated presence of cells from only the most dorsal parts of the subpallium structure (LGE, CGE) and absence of ventrally located medial ganglionic eminence (MGE) cells, which is consistent with the previously mentioned lack of *NKX2.1* expression. We also noticed a very small group of cells (less than <1 %) that demonstrated strong expression of *OTX2*, *LMX1a*, and *RSPO2 *(Figure 4o(Fig. 4) and S4o and S5o), which confirmed presence of cortical hem cells in the COs, in line with the immunostaining results. 

We also quantified the distribution of cells in different clusters, to compare the cellular composition of the COs at two differentiation time points, day 80 and day 160 (Figure 4p(Fig. 4) and S4p and S5p). By transitioning from day 80 to day 160 of differentiation, the neuronal composition of the COs changed. There was an increase in the proportion of inhibitory interneurons (INs) compared to glutamatergic excitatory neurons (ExNs), besides the explicit increase in the number of emerging astrocytes and oligodendrocytes (Glia) over this period. Nevertheless, the neural progenitor (NPs) cell content remained at a quite constant ratio between day 80 and day 160, indicating the sustained presence of neural progenitor cells in the COs. 

Dissecting the identity of the different cell clusters and expression patterns of different cell type-specific genes illustrated a similar layout in the COs derived from different hPSCs (Figure 4(Fig. 4) and S4 and S5). However, this similarity was more evident at day 160, the more mature stage of COs. Indeed, at the earlier time point (day 80), COs derived from different hPSCs displayed different degrees of maturation and consequently, contained dissimilar proportions of late-born populations, e.g., inhibitory interneurons and glial cells. 

Thus, the histological and gene expression analysis demonstrated that the cellular composition of the COs simulated diverse cortical neurons and glial cell identities that matched the dorsal-forebrain region and also the more dorsally located ganglionic eminences (LGE, CGE) in the subpallium region of the developing cortex (Figure 4q(Fig. 4)). It should be noted that identities like blood vessels and microglia were absent from the organoids, in line with the fact that we blocked signaling pathways towards non-ectodermal lineages differentiation during the first step of the protocol.

### Neurons of cerebral organoids are electrically functional and display network conduction

**Figure 5:**
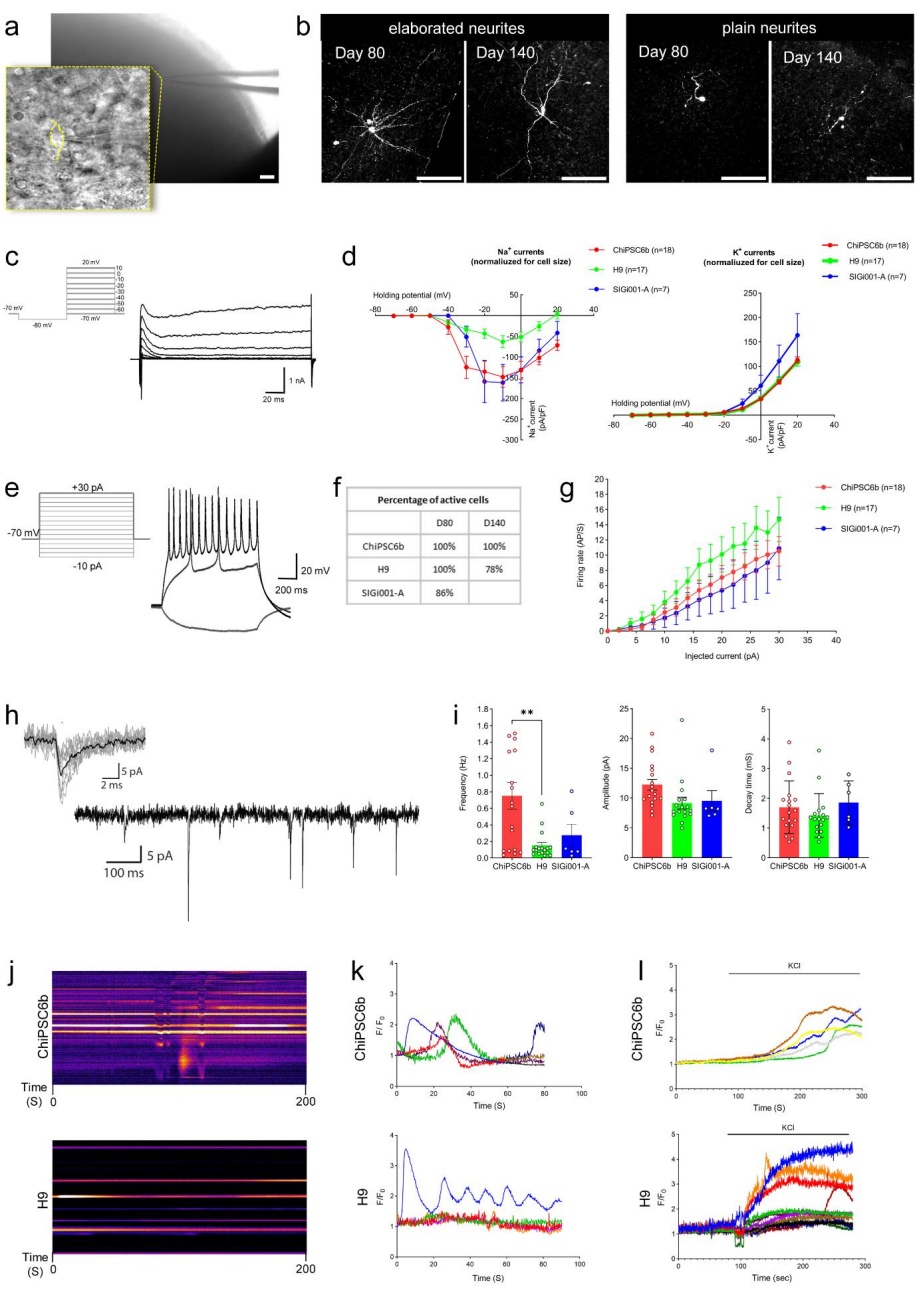
Functional properties of neurons and network activity of cerebral organoids. (a) Bright-field image of recorded neurons within sliced COs. (b) Biocytin staining of patched slices demonstrating the presence of neurons with various neurites complexity within both days 80 and 140. Scale bars are 100 μm. (c) Representative trace of Na^+^/K^+^ current measurement and (d) I-V curve of Na^+^ and K^+^ channel currents in COs. (e) Representative trace for action potential profiling (in current clamp). (f) Percentage of patched neurons which were able to fire induced-action potential within sliced COs. (g) The firing rate of action potential showed that neurons in COs are electrophysiologically active. (h) Sample recording trace of spontaneous excitatory synaptic input (sEPSCs) and (i) related quantified characteristics (frequency, amplitude, and decay time). The number of patched neurons related to COs derived from different pluripotent cell lines is illustrated within each graph (mean ± SEM; one-way ANOVA, **p < 0.01). (j) Visual representation of intracellular Ca^2+^ measurements in Fluo-4 AM loaded COs derived from ChiPSC6b and H9 cell lines, on day 160, showing a time-lapse of the first 200 seconds obtained after re-slicing the image stack, using ImageJ software, of COs across a straight line drawn across spontaneously active neurons. (k) Typical Ca^2+^ traces representing spontaneous Ca^2+^ release and (l) KCl-induced depolarization followed by Ca^2+^ entry. Regions of interest were drawn on responding cells of the respective organoid. All traces are depicted as F/F_0_.

To evaluate electrophysiological characteristics, we sectioned day 80 and 140 COs into 300 µm slices and performed patch clamp recordings of individual neurons (Figure 5a(Fig. 5)). During whole-cell recordings, we subjected the recorded neurons to biocytin labeling infused in intracellular medium, to make the neurites visible (Figure 5b(Fig. 5)). We observed that the different patched neurons within COs, on both day 80 and day 140, exhibited two general types of neurites branching complexity, including plain and also more elaborate neurites. We explored some intrinsic electrophysiological features such as resting membrane potential, input resistance, and action potential threshold in COs derived from different hPSC lines (Figure S6a). As there was no significant difference between day 80 and day 140 measurements, we grouped the values of recorded neurons based on which hPSC line they were derived from. The Na^+^ and K^+^ currents were measured in a voltage clamp, with incremental depolarizing steps from -70 to +10 mV, to elicit Na^+^ and K^+^-channel activation. The COs derived from H9 hESC line exhibited smaller sodium and potassium currents in comparison to ChiPSC6b and SIGi001-A hiPSC lines, even though their difference is significant only for Na^+^ currents (Figure 5c,d(Fig. 5)). Almost all recorded neurons fired action potentials in response to a step-by-step increase in current injections (Figure 5e,f(Fig. 5)). In this assessment action potential firing rate (AP/S) represents the maturation profile of the neurons, as more current is required to activate more mature neurons, and the total amount of APs increases during later and higher stimulations. The H9 hESC line-derived COs displayed a less mature AP profile, compared to the ChiPSC6b and SIGi001-A hiPSC lines-derived COs, but not in a significant way (Figure 5g(Fig. 5)). Besides, quantified properties of the action potentials were comparable for COs derived from different hPSCs, reflecting that the overall maturation extent of their neurons is similar (Figure S6b). Moreover, spontaneous action potentials were measured at the resting membrane potential of the cells. In COs of both ages, neurons were mature enough to fire spontaneous action potentials, which is expected because the average resting membrane potential is around the action potential threshold (~-40 mV) (Figure S6c). Furthermore, we evaluated spontaneous excitatory synaptic input (sEPSCs) into recorded neurons using voltage clamp recordings (Vm = -70 mV) (Figure 5h(Fig. 5)). ChiPSC6b-derived COs showed more spontaneous input compared to H9 and SIGi001-A-derived COs. The amplitudes and decay time were comparable between all hPSC lines-derived COs (Figure 5i(Fig. 5)). There was spontaneous input in all recorded cells, indicating that neurons in the COs are capable of getting involved in network conduction. 

To assess the ability of neurons to behave as a synchronized network in COs, the intracellular Ca^2+^ dynamics were monitored in whole CO after loading the fluorescent Ca^2+^ indicator dye, Fluo-4 AM, in the medium. Spontaneous intracellular Ca^2+^ releases were detected over the monitoring time. This spontaneous activity is a consequence of the occurrence of action potentials triggering Ca^2+^ influx over the plasma membrane and Ca^2+^ release from intracellular stores in the neuronal cells (Figure 5j,k(Fig. 5)). Additionally, potassium chloride (KCl) treatment was used to induce depolarization in Fluo-4 AM-loaded COs (Figure 5l(Fig. 5)). Upon Ca^2+^ entry, the Fluo-4 signal clearly shows axonal/dendritic connections between the different cells within the organoid (Supplementary Video 1). Accordingly, neurons within the COs displayed electrical conduction through a synchronized network. 

Altogether, the electrophysiological features and neuronal functionality of COs derived from different hPSCs, were quite similar and also comparable to recorded values from the human developing brain (Moore et al., 2009[29]) and previously reported studies (Watanabe et al., 2017[57]).

## Discussion

Despite the numerous published protocols on the production of different types of neural organoids from hPSCs, there are still significant shortcomings in the way of utilizing these organoids as a widely applicable and reliable model to investigate cellular and molecular human-specific neurological events. The differentiation of hPSCs to neural fates and progenies can manifest variable outcomes under *in vitro* cell culture conditions.

In this study, by considering some of the main underlying contributors to these variable results, we described a simple protocol to generate COs from hPSCs. This method is based on previously published "guided" protocols but with a more comprehensive small molecule and growth factor formulation, to ensure achievement of anterior-dorsal neural fates of the cerebral cortex. In contrast to other studies, we did not use ultra-low attachment plates for the aggregate formation step but used a modified hanging drop method. The dynamic culture condition was provided on an orbital shaker which, compared to using spinner flasks, requires less culture medium for long-term CO culture. In addition, our protocol did not include the laborious procedure of embedding organoids into Matrigel droplets. All the above, make this method a more facile and affordable way to generate COs in most laboratories.

Formation of the VZ-like structures is one of the unique features of neural organoids, which provides a dynamic pool of neural progenitor cells within COs to generate diverse neural cell populations in the *in vivo*-like spatiotemporal pattern. The induced neural progenitor cells form epithelial structures, first at the periphery of the organoids, afterward, they gain apical-basal polarity based on sensing basement membrane proteins on the apical side, then undergo a rolling morphogenetic movement to construct the VZ-like organization inside the organoids (Andrews et al., 2022[2]; Nasu et al., 2012[30]). Therefore, exposing organoids to an extracellular matrix can expedite the VZ-like structure formation and this is the main reason for using Matrigel in most protocols of neural organoid generation. Nevertheless, as was also shown in protocols published by the Pasca group (Sloan et al., 2018[43]), we demonstrated that these structures can be generated without adding any exogenous extracellular matrix and only based on the presence of basement membrane proteins produced by the neural cells endogenously. Moreover, different lots of Matrigel, extracted from a mouse cancer cell line, contain different concentrations of ECM proteins and growth factors (Hughes et al., 2010[16]), which may affect neural differentiation outcome. Therefore, we excluded Matrigel from our protocol and include CHIR99021 and heparin treatment instead, to boost WNT signaling that has been shown to make VZ-like structures larger (Lancaster et al., 2017[25]). 

In the context of 3D suspension culture conditions, organoids sense no biochemical micropatterning guidance or biophysical staddle, like what is provided by the notochord during *in vivo* development (Corallo et al., 2015[9]; Stemple, 2005[45]). Consequently, there is an inevitable heterogeneity in VZ-like structure size and localization as well as the organization of different neural cells. Therefore, to have a comparable criterion between different CO samples, studies used single-cell transcriptome to assess the cell-type composition and diversity within organoids. We also used snRNA-seq analysis to evaluate the cellular combination of COs derived from hPSC lines. A diverse cell population, including neural progenitors, deep and upper cortical layer neurons was identified within our COs, and more importantly, this diversity increased at the later time point (day 160 COs) due to the emergence of inhibitory interneurons, astrocytes, and oligodendrocytes. This cell-type configuration is comparable to the single-cell RNA sequencing results of other studies which generated forebrain organoids (Qian et al., 2020[38]; Sivitilli et al., 2020[42]; Tanaka et al., 2020[49]; Velasco et al., 2019[53]). A substantial fraction of these dorsal-forebrain organoid cell identities is allocated to inhibitory interneurons, despite the known ventral origin of these cell types. Likewise, the presence of inhibitory interneurons is reported in the majority of CO generation protocols, even if no ventralizing treatments, such as SHH signaling pathway activators, were used (Tanaka et al., 2020[49]). In fact, some partial ventralization can occur during CO differentiation, even without using any extrinsic SHH activator (Yoon et al., 2019[60]). This is in agreement with detecting the molecular signatures of only the more dorsal parts (lateral and caudal) of ganglionic eminences and not the most ventral (medial) part of ganglionic eminences. Additionally, this also can be explained by considering the reports that imply a dorsal origin for a portion of GABAergic interneurons in primates, contrary to rodents in which the interneurons originate just from subpallium parts of the developing brain (Petanjek et al., 2009[35]; Yu and Zecevic, 2011[62]). 

From a functional point of view, electrophysiological properties of patched neurons within acute sliced COs as well as Ca^2+^ handling studies indicated that neurons are mature enough to display electrical and synaptic activity and can behave as a network, both spontaneously and in response to a stimulus. Nonetheless, the recorded values of neuronal activity in the COs are still far from the electrophysiological features of more mature neurons related to post-natal stage of human brain. This is in line with other studies, which demonstrated that functional maturation profile of neurons in COs reaches to mid-gestational age of developing human brain (Moore et al., 2009[29]; Watanabe et al., 2017[57]). 

In conclusion, we aimed to devise a straightforward differentiation method for generating COs from hPSCs. These COs comprise different types of functional neurons and glial cells, organized in a rudimentary *in vivo*-like arrangement. Along with taking advantage of establishing hiPSCs from patients or engineering a disease-specific genetic background, hiPSC-derived COs provide a reductionist model to determine the key pathways or master regulators underlying a super complex network affected by disease or developmental processes and also to discover the best possible target in drug screening.

## Notes

Yoke Chin Chai, Catherine Verfaillie (Stem Cell Institute, Department of Development and Regeneration, KU Leuven, Leuven 3000, Belgium; E-mail: catherine.verfaillie@kuleuven.be) and Hossein Baharvand (Department of Stem Cells and Developmental Biology, Cell Science Research Center, Royan Institute for Stem Cell Biology and Technology, ACECR, Tehran, Iran; E-mail: h.baharvand@royan-rc.ac.ir) contributed equally as corresponding author.

## Declaration

### Acknowledgment

We would like to thank Mostafa Najar-Asl, Hassan Ansari, Fatemeh Arefeh Nami, and Niels Vidal for their consultation and technical assistance. We are also grateful to Youssef El Laithy for providing us with the WNT2B antibody. 

### Funding

This work was financially supported by KU Leuven Stem Cell Institute, and Royan Institute for Stem Cell Biology and Technology and the Cognitive Science and Technologies Council of Iran. SS, Y-CC, and CV were supported by the Research Foundation - Flanders (FWO) (FWO-SBO-S001221N-OrganID), TV is a recipient of a FWO post doc fellowship (12ZG121N) the Belgium Alzheimer's Research Foundation (SAO-#3M200141), the America Alzheimer's Association (AARG-NTF-19-616888), KUL C14/17/111 -3D-MuSYC, and the Mitialto Foundation. Research in the Pasque laboratory was supported by the Research Foundation-Flanders (FWO; Odysseus Return Grant G0F7716N to VP; FWO grants G0C9320N and G0B4420N to VP), the KU Leuven Research Fund (C1 grant C14/21/ 19 to VP), and FWO PhD fellowships to SKT (1S75720N).

### Conflict of interest

The authors declare that they have no conflict of interest.

### Ethics approval

All methods and experiments using hESCs/hiPSCs were conducted in accordance with the approved guidelines of Ethics Committee at the University Hospital, Gasthuisberg, KU Leuven, Belgium and the Institutional Review Board and Ethical Committee of Royan Institute, Tehran, Iran (IR.ACECR.ROYAN.REC.1398.96).

### Consent to participate

Not applicable.

### Consent for publication

Not applicable.

### Availability of data and material

All generated and analyzed data of this study are available from the lead contact on reasonable request. Single-nucleus transcriptome data is deposited to GEO database, BioProject ID PRJNA935156.

### Authors' contributions

The study design was performed by Hossein Baharvand, Catherine Verfaillie, and Susan Simorgh. Material preparation, data collection and analysis were performed by Susan Simorgh, Seyed Ahmad Mousavi, San Kit To, Keimpe Wierda, Tim Vervliet, Yoke Chin Chai, Meghdad Yeganeh, Paria Pooyan. Vincent Pasque [and others] designed the snRNA-seq experiment. The first draft of the manuscript was written by Susan Simorgh. Catherine Verfaillie, Hossein Baharvand, Yoke Chin Chai, and Meghdad Yeganeh, commented on previous versions of the manuscript. All authors read and approved the final manuscript.

## Supplementary Material

Supplementary information

Supplementary video

## Figures and Tables

**Figure 1:**
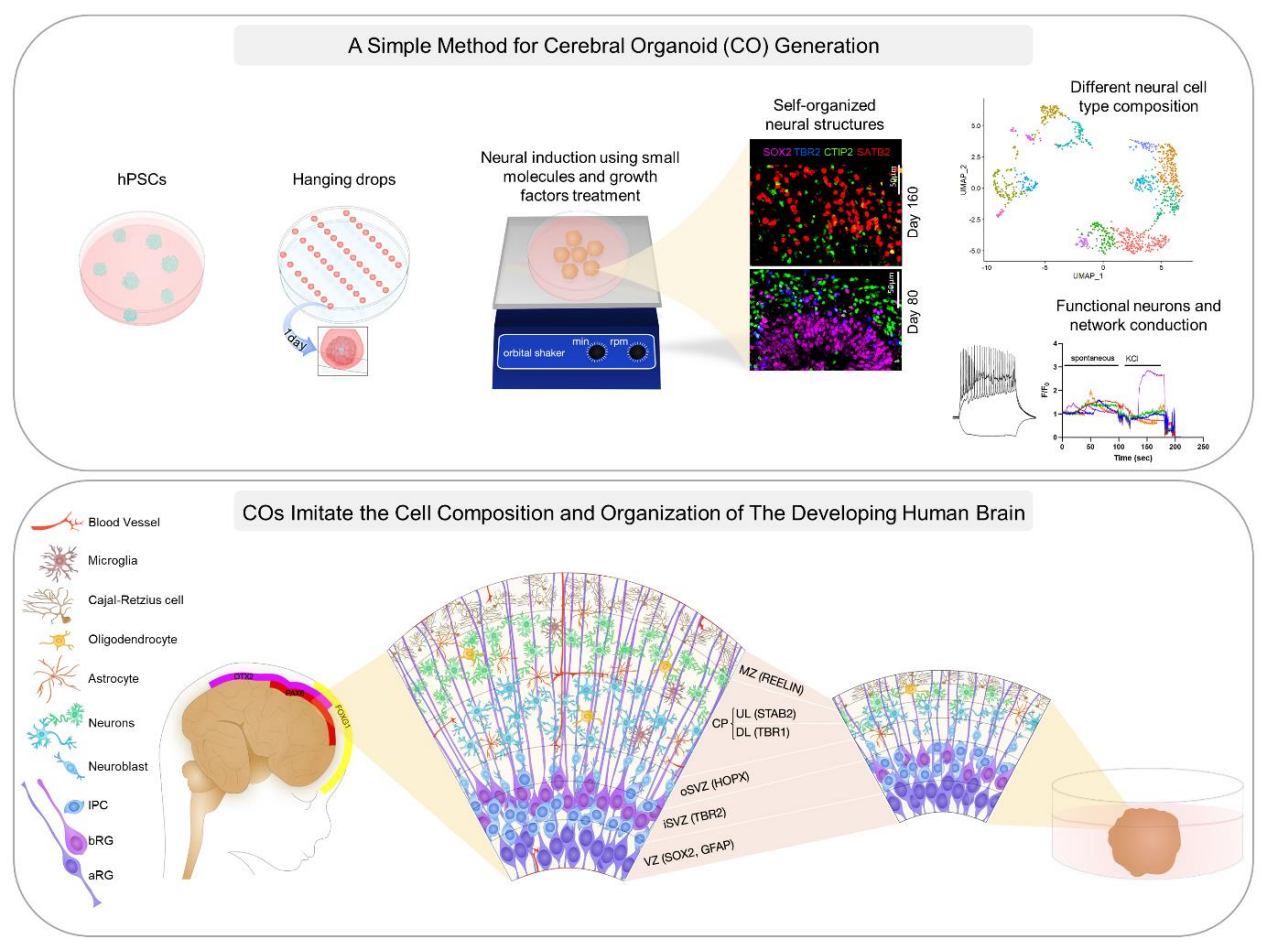
Graphical abstract
