# Supplementary material for: A facile method to generate cerebral organoids from human pluripotent stem cells
Source: EXCLI J. 2023 Oct 5;22:1055–76. doi: 10.17179/excli2023-6299 (PMC10620858; doi:10.17179/excli2023-6299)
Supplement: Supplementary information [file EXCLI-22-1055-s-001.pdf]

EXCLI Journal 2023;22:1055-1076 – ISSN 1611-2156
Received: July 04, 2023, accepted: September 26, 2023, published: October 05, 2023

Supplementary information to:

Original article:

A FACILE METHOD TO GENERATE CEREBRAL ORGANOIDS FROM HUMAN PLURIPOTENT STEM CELLS

Susan Simorgh[1,2,3], Seyed Ahmad Mousavi[2], San Kit To[4], Vincent Pasque[4], Keimpe Wierda[5,6], Tim Vervliet[7], Meghdad Yeganeh[2], Paria Pooyan[2], Yoke Chin Chai[3*], Catherine Verfaillie[3*], Hossein Baharvand[1,2*]

1 Department of Developmental Biology, School of Basic Sciences and Advanced Technologies in Biology, University of Science and Culture, Tehran, Iran
2 Department of Stem Cells and Developmental Biology, Cell Science Research Center, Royan Institute for Stem Cell Biology and Technology, ACECR, Tehran, Iran
3 Stem Cell Institute, Department of Development and Regeneration, KU Leuven, Leuven 3000, Belgium
4 Department of Development and Regeneration, Lab for Epigenetic Reprogramming, Leuven Stem Cell Institute, Leuven Single-Cell Omics Institute and Leuven Cancer Institute, KU Leuven-University of Leuven, Leuven 3000, Belgium
5 VIB-KU Leuven Center for Brain & Disease Research, Leuven 3000, Belgium
6 Electrophysiology Unit, Leuven 3000, Belgium
7 Laboratory of Molecular and Cellular Signaling, Department of Cellular and Molecular Medicine, KU Leuven, Leuven 3000, Belgium

* **Corresponding authors:** Hossein Baharvand, Department of Stem Cells and Developmental Biology, Cell Science Research Center, Royan Institute for Stem Cell Biology and Technology, ACECR, Tehran, Iran; E-mail: h.baharvand@royan-rc.ac.ir
Catherine Verfaillie, Stem Cell Institute, Department of Development and Regeneration, KU Leuven, Leuven 3000, Belgium; E-mail: catherine.verfaillie@kuleuven.be
Yoke Chin Chai, Stem Cell Institute, Department of Development and Regeneration, KU Leuven, Leuven 3000, Belgium; E-mail: yokechin.chai@kuleuven.be

https://dx.doi.org/10.17179/excli2023-6299

This is an Open Access article distributed under the terms of the Creative Commons Attribution License (http://creativecommons.org/licenses/by/4.0/).

EXCLI Journal 2023;22:1055-1076 – ISSN 1611-2156
Received: July 04, 2023, accepted: September 26, 2023, published: October 05, 2023

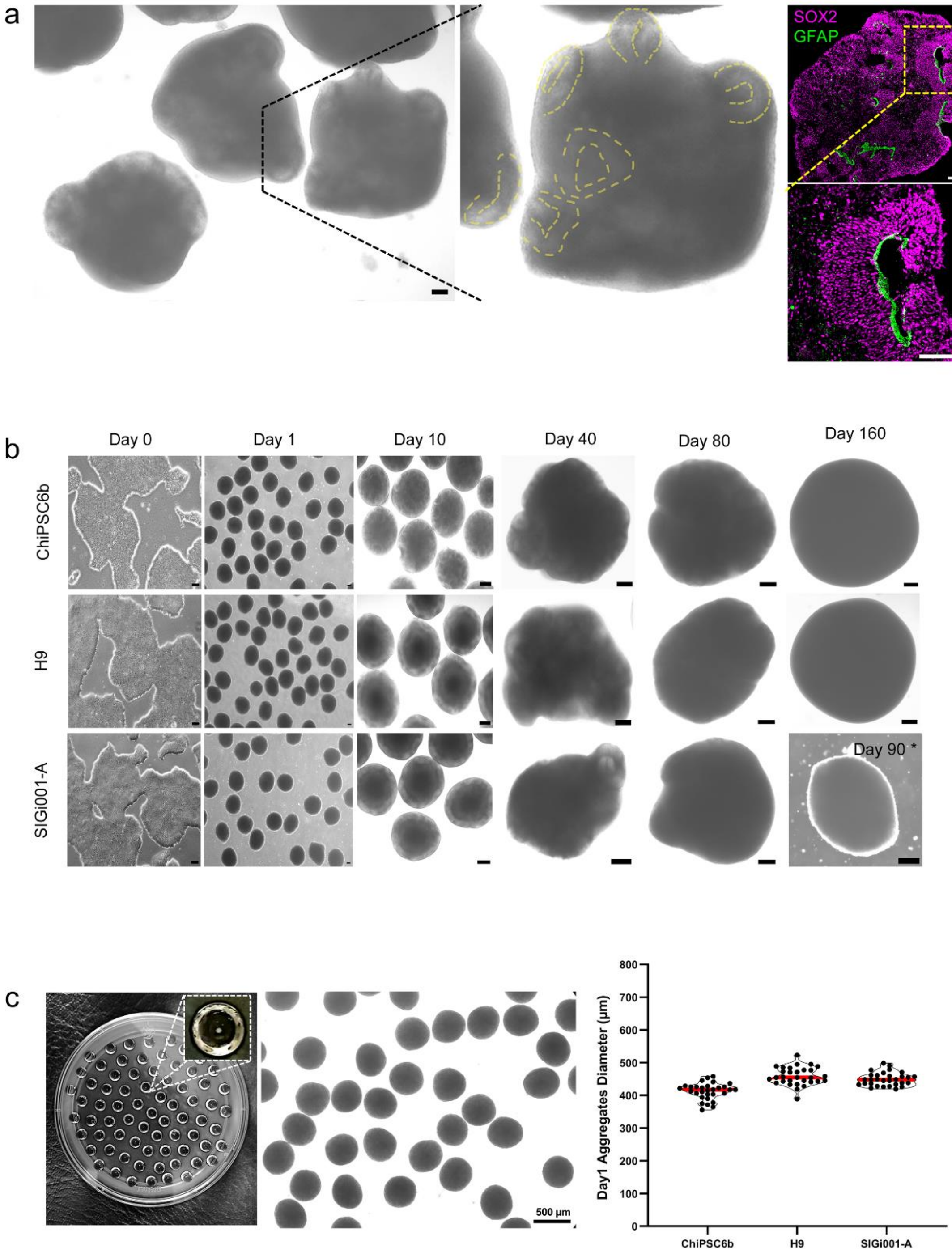

(Supplementary) Figure S1: Generation of cerebral organoids from three human pluripotent cell lines (ChiPSC6b-hiPSC, H9-hESC, SIGi001-A-hiPSC). (**a**) Formation of multiple VZ-like structures within the COs between days 35 to 40. These structures encompassed a VZ-like cavity space that is surrounded by radially organized neural progenitors and depicted by SOX2/GFAP immunostaining. (**b**) Bright-field images showing morphological changes of COs derived from different human pluripotent cell (hPSC) lines during different days of the protocol. * COs derived from SIGi001-A-hiPSC showed extensive cell death around day 90 and did not survive until day 160.
All the scale bars are 100 µm.
(**c**) Aggregate formation using the modified hanging drop method led to generation of uniform spherical structures with a diameter size between 350 to 450 µm from hPSC lines on day 1. (The red median line in the graph indicates the average size of the aggregates.)

EXCLI Journal 2023;22:1055-1076 – ISSN 1611-2156
Received: July 04, 2023, accepted: September 26, 2023, published: October 05, 2023

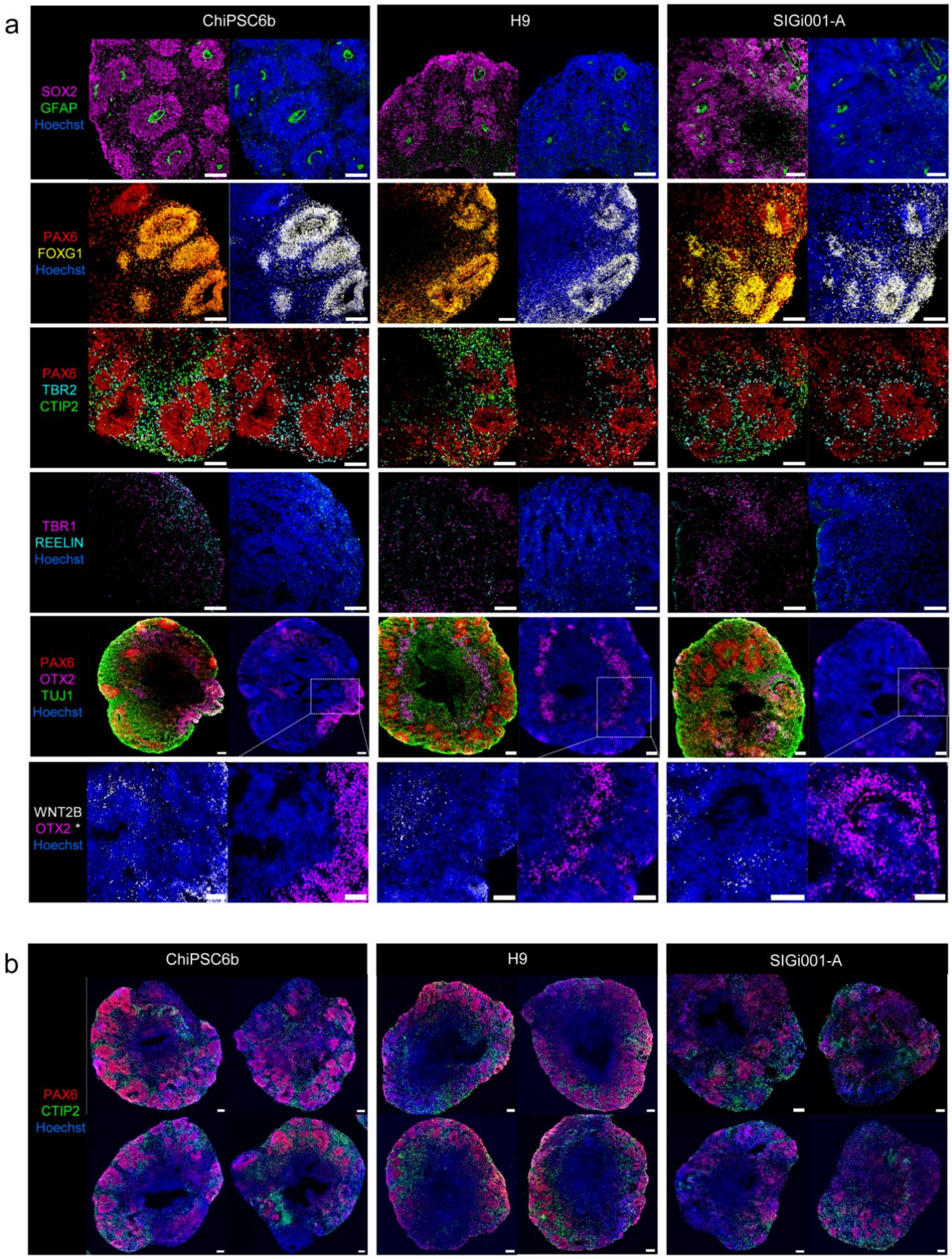

Figure S2: Immunostaining analysis to investigate different cell type identities and organization in day 40 cerebral organoids derived from three human pluripotent cell lines (ChiPSC6b-hiPSC, H9-hESC, SIGi001-A-hiPSC). (**a**) Representative immunostaining images indicating similar expression patterns of specific markers for positional neural cell fates (PAX6/FOXG1, OTX2, and WNT2B), ventricular-like zone (SOX2/GFAP), and early cortical neuron layering organization (TBR2/CTIP2 and TBR1/REELIN) on day 40, in different hPSC lines derived COs. (**b**) Sample immunostaining images from different hPSC lines derived COs on day 40, demonstrating cytoarchitecture of VZ-like regions (PAX6) and early cortical neurons layer (CTIP2).
All the scale bars are 100 µm.

EXCLI Journal 2023;22:1055-1076 – ISSN 1611-2156
Received: July 04, 2023, accepted: September 26, 2023, published: October 05, 2023

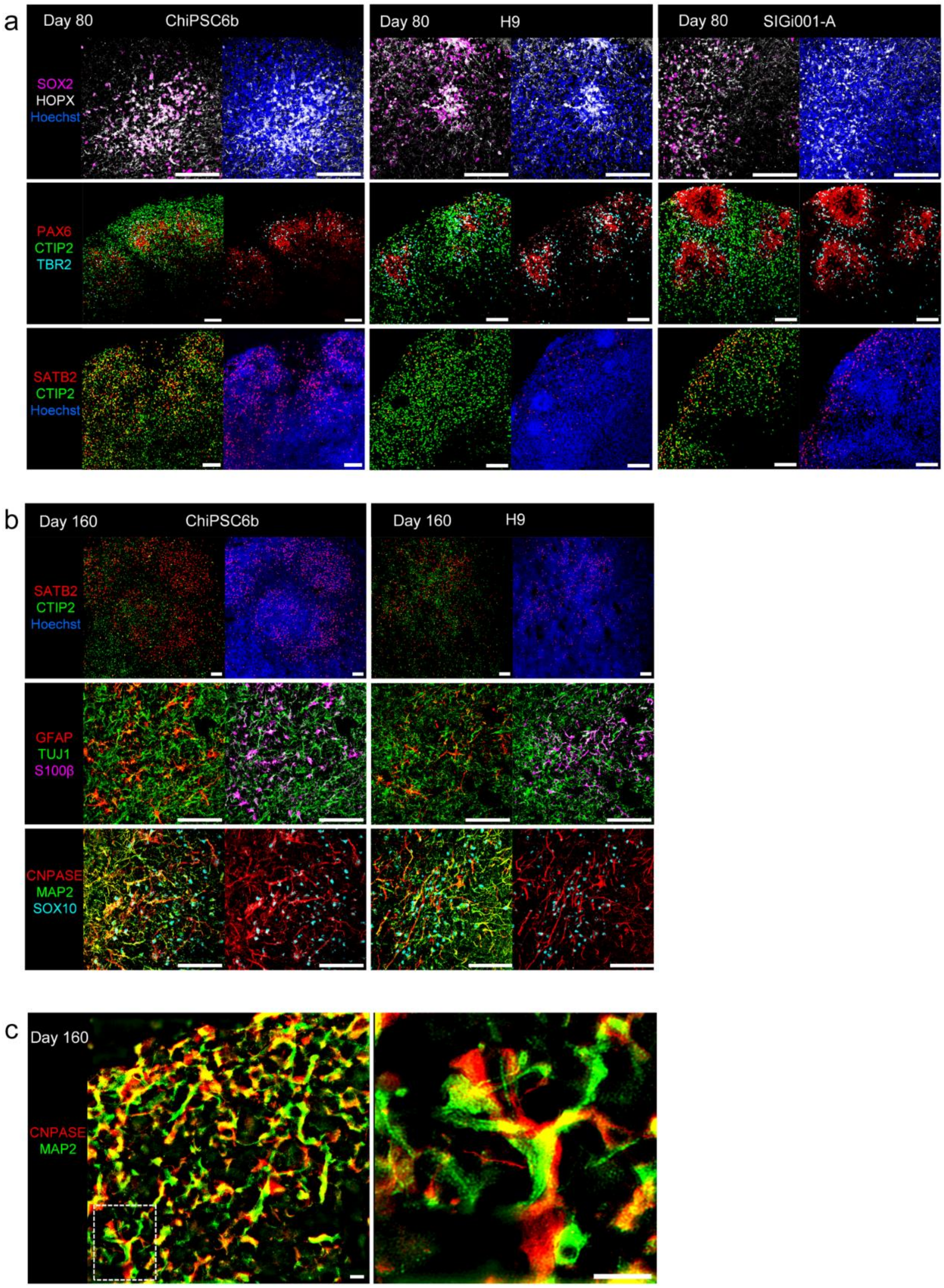

Figure S3: Overview of expression pattern for maturation indicating markers in cerebral organoids derived from different hPSC lines. (**a**) Immunostaining analysis for the presence of basal radial glial cells (SOX2/HOPX), intermediate progenitor cells (TBR2), and deep and superficial cortical layer neurons (CTIP2/SATB2) on day 80. (**b**) Day 160 COs stained for deep and superficial cortical layer neurons (CTIP2/SATB2), astrocytes (GFAP/S100β), and oligodendrocytes (CNPASE/SOX10) markers indicating maturation progress in COs derived from different hPSC lines. (**c**) Higher magnifications of immunostaining for oligodendrocyte marker (CNPASE) illustrated these cells in close contact with neurite extensions (MAP2).
All the scale bars are 100 µm.

EXCLI Journal 2023;22:1055-1076 – ISSN 1611-2156
Received: July 04, 2023, accepted: September 26, 2023, published: October 05, 2023

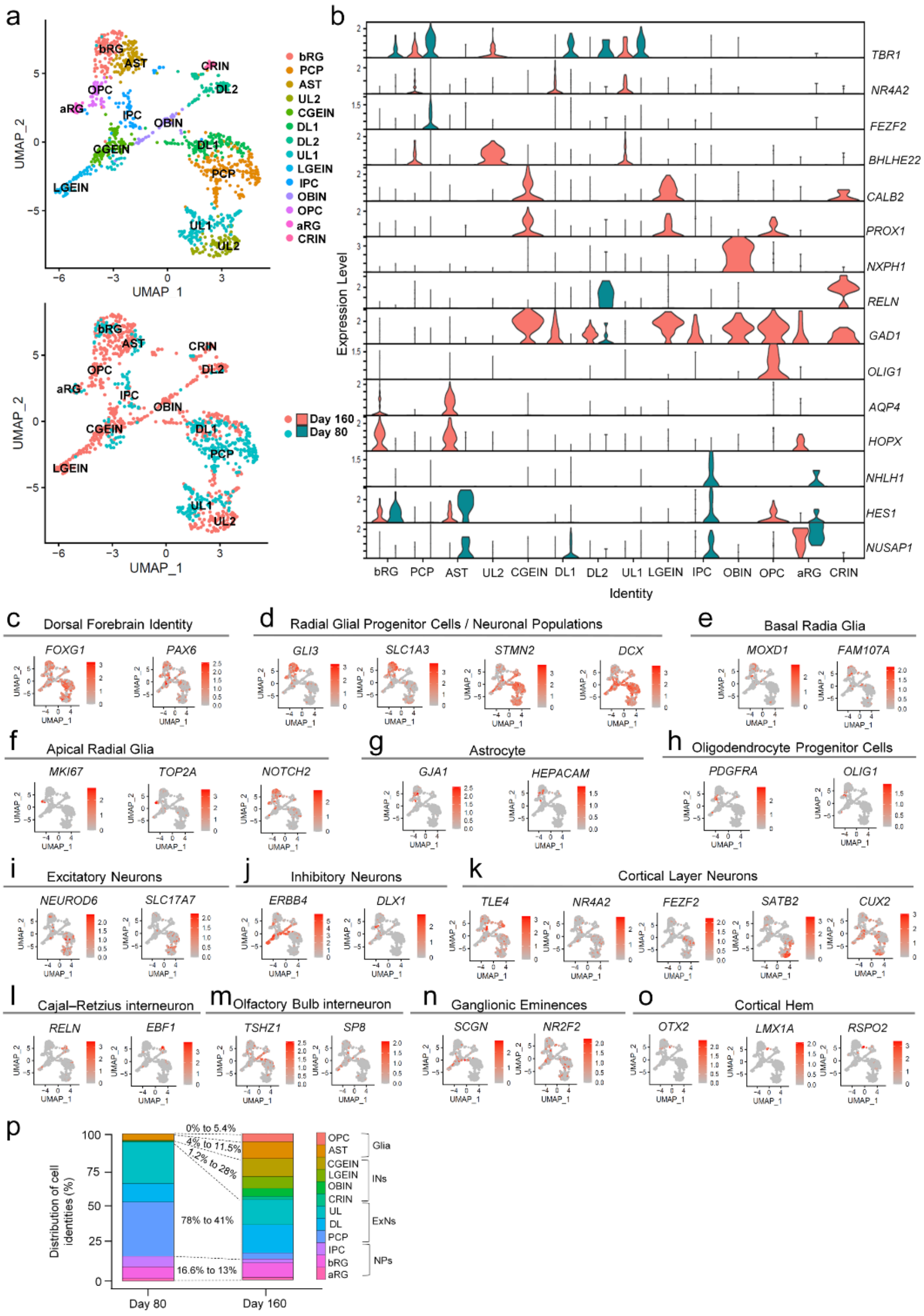

Figure S4: Determining the cell composition of H9 hPSC-COs by snRNA-seq analysis. (**a**) Uniform-manifold approximation map (UMAP) visualization of different cell-type clusters in COs derived from H9 cell line. UMAPs are represented as integrated data from day 80 and day 160 with n=447 and n=823 nuclei analyzed for each day, respectively. Each dot represents a single nucleus from a single cell. In the upper UMAP, the color of each dot entitles the defined cell identities, and in the lower UMAP colors illustrate the corresponding day. (**b**) Violin plots demonstrating the expression level of selected specific markers to ascertain different cell identities in each cluster. (**c-o**) Individual UMAP plots, indicating the expression pattern of marker genes used to annotate a defined cell identity for each cluster. The gradient color shows the relative expression pattern from dark orange (higher expression) to light gray (lower expression). (**p**) Bar graphs illustrating the percentage of cellular distribution in different clusters, on days 80 and 160 COs. The colors label the cell type identities in the graphs.
(aRG: apical radial glial, bRG: basal radial glial, AST: astrocyte, OPC: oligodendrocyte progenitor cell, PCP: primary cortical plate, DL1,2: Deep layer cortical neuron 1,2, UL1,2: Upper layer cortical neuron 1,2, CGEIN: caudal ganglionic eminences interneuron, LGEIN: lateral ganglionic eminences interneuron, CRIN: Cajal–Retzius interneuron, OBIN: olfactory bulb interneuron, NPs: neural progenitor cells, IPC: intermediate progenitor cell, ExNs: excitatory neurons, INs: inhibitory neurons)

EXCLI Journal 2023;22:1055-1076 – ISSN 1611-2156
Received: July 04, 2023, accepted: September 26, 2023, published: October 05, 2023

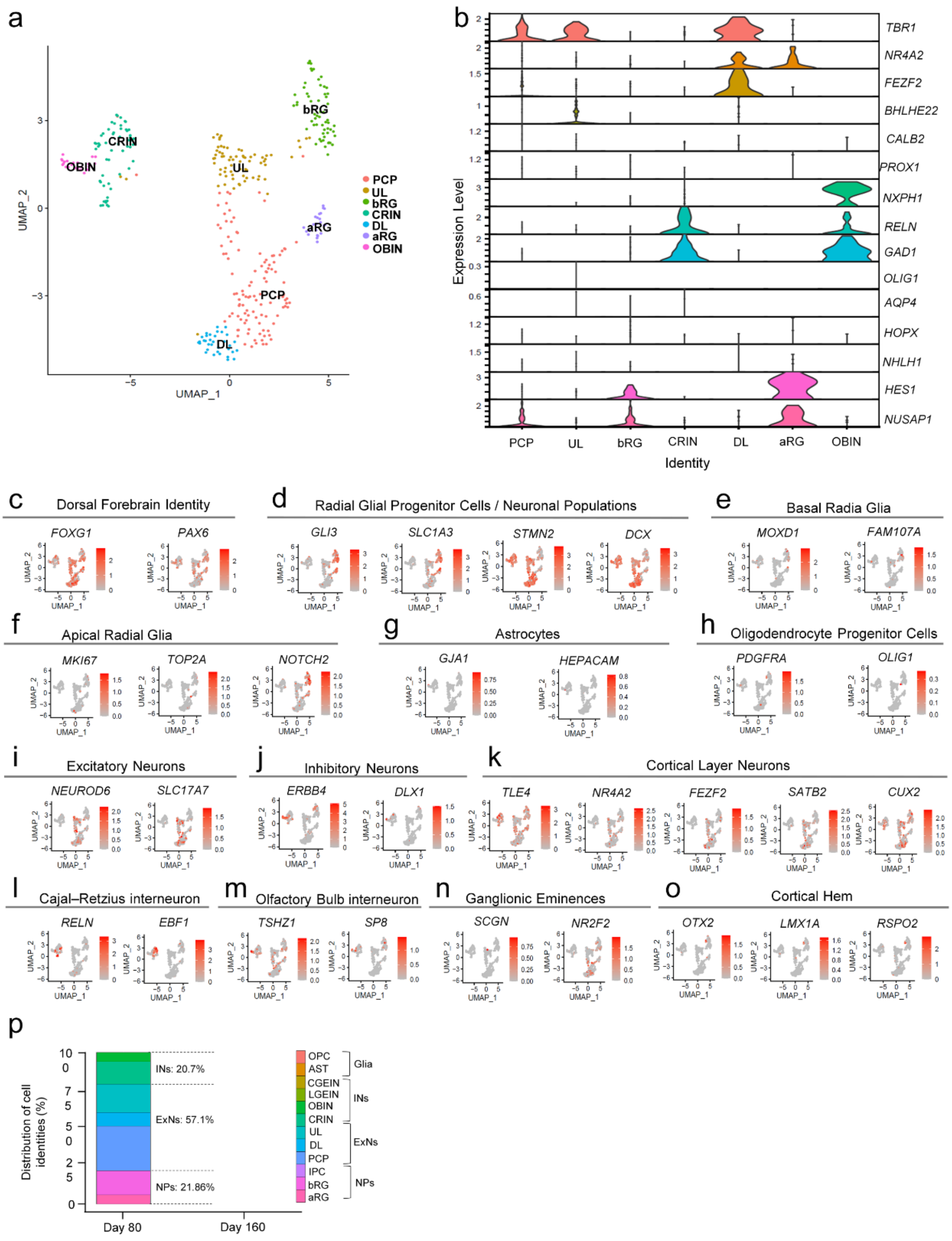

Figure S5: Determining the cell composition of SIGi001-A hPSC-COs by snRNA-seq analysis. (**a**) UMAP illustration of snRNA-seq data analysis of n=379 nuclei from day 80 COs derived from SIGi001-A hPSC line. The color of each dot entitles the defined cell identities of each analyzed nucleus. (**b**) Violin plots demonstrating the expression level of selected specific markers to ascertain different cell identities in each cluster. (**c-o**) Individual UMAP plots, indicating the expression pattern of marker genes used to annotate a defined cell identity for each cluster. The gradient color shows the relative expression pattern from dark orange (higher expression) to light gray (lower expression). (aRG: apical radial glial, bRG: basal radial glial, AST: astrocyte, OPC: oligodendrocyte progenitor cell, PCP: primary cortical plate, DL1,2: Deep layer cortical neuron 1,2, UL1,2: Upper layer cortical neuron 1,2, CGEIN: caudal ganglionic eminences interneuron, LGEIN: lateral ganglionic eminences interneuron, CRIN: Cajal–Retzius interneuron, OBIN: olfactory bulb interneuron, NPs: neural progenitor cells, IPC: intermediate progenitor cell, ExNs: excitatory neurons, INs: inhibitory neurons)

EXCLI Journal 2023;22:1055-1076 – ISSN 1611-2156
Received: July 04, 2023, accepted: September 26, 2023, published: October 05, 2023

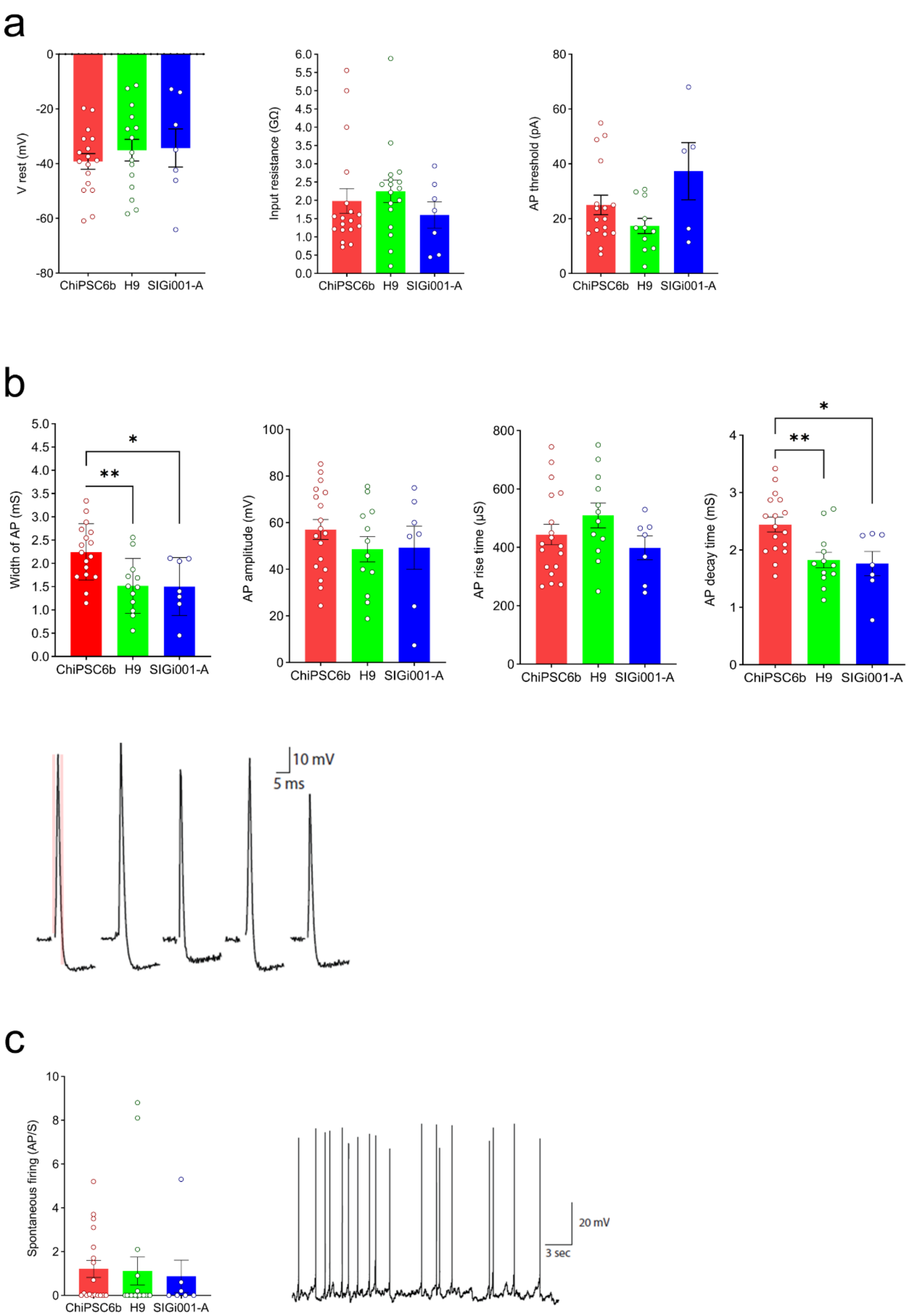

Figure S6: Electrophysiological analysis of cerebral organoids. (**a**) Measured intrinsic physiological properties of neurons including resting membrane potential, input resistance, and action potential threshold. (**b**) Single action potential parameters were measured for COs derived from different pluripotent cell lines. (**c**) Spontaneous action potential firing and related sample recorded trace. The number of neurons related to each pluripotent cell line-derived CO is depicted within each graph. All data represented as mean ± SD; one-way ANOVA, $*p < 0.05$, $**p < 0.01$).

EXCLI Journal 2023;22:1055-1076 – ISSN 1611-2156
Received: July 04, 2023, accepted: September 26, 2023, published: October 05, 2023

Supplementary Video 1: KCl-induced depolarization followed by Ca^{2+} entry in Fluo-4 AM loaded cerebral organoid. See file https://doi.org/10.17179/excli2023-6299/supplementary_video

Supplementary Table 1: List of primary and secondary antibodies used for immunostaining

Antigen	Company	Cat. no.	Dilu-tion
**Rabbit anti-SOX2**	Millipore	AB5603	1:100
**Rat anti-GFAP**	Thermo Fisher Scientific	0300-13	1:200
**Mouse anti-PAX6**	Abcam	ab78545	1:100
**Rabbit anti-FOXG1**	Abcam	ab196868	1:100
**Rabbit anti-OTX2**	Abcam	ab21990	1:100
**Chicken anti-TUJ1**	Millipore	AB9354	1:200
**Rabbit anti-WNT2B**	Abcam	ab178418	1:100
**Rabbit anti-TBR2**	Abcam	ab23345	1:100
**Rat anti-CTIP2**	Abcam	ab18465	1:100
**Rabbit anti-TBR1**	Abcam	ab31940	1:100
**Mouse anti-REELIN**	Abcam	ab78540	1:100
**Mouse anti-HOPX**	Santa Cruz Biotechnology	sc-398703	1:50
**Rabbit anti-S100B**	Abcam	ab52642	1:100
**Rabbit anti-MAP2**	Synaptic Systems	002188	1:200
**Goat anti-SOX10**	R&D Systems	AF2864	1:100
**Mouse anti-CNPASE**	Sigma	C5922	1:100
**Mouse anti-SATB2**	Abcam	ab51502	1:100
**NorthernLights Streptavidin NL557**	R&D Systems	NL999	1:200
**Donkey anti-Rabbit, Alexa Fluor 647**	Thermo Fisher Scientific	A-31573	1:500
**Donkey anti-Rat, Alexa Fluor 488**	Thermo Fisher Scientific	A-21208	1:500
**Donkey anti-Mouse, Alexa Fluor 555**	Thermo Fisher Scientific	A-31570	1:500
**Goat anti-Chicken, Alexa Fluor 488**	Thermo Fisher Scientific	A-11039	1:500
**Donkey anti-Rabbit, Alexa Fluor 488**	Thermo Fisher Scientific	A-21206	1:500
**Donkey anti-Goat, Alexa Fluor 633**	Thermo Fisher Scientific	A-21082	1:500
